# Delayed Traumatic Tricuspid Valve Regurgitation Caused by Blunt Chest Trauma: A Case Report and Literature Review

**DOI:** 10.7759/cureus.73001

**Published:** 2024-11-04

**Authors:** Reda Mounir, Fouad Nya, Noureddine Atmani, Abdessamad Abdou, Youness Moutakillah

**Affiliations:** 1 Department of Cardiothoracic Surgery, Avicenna Military Hospital, Cadi Ayyad University, Marrakech, MAR; 2 Department of Cardiovascular Surgery, Mohammed V Military Hospital, Mohammed V University, Rabat, MAR; 3 Department of Cardiovascular Surgery, Faculty of Medicine and Pharmacy of Fes, Sidi Mohammed Ben Abdellah University, Fes, MAR; 4 Department of Cardiac Surgery, Mohammed V Military Teaching Hospital, Mohammed V University, Rabat, MAR

**Keywords:** chest trauma, echocardiography, traumatic valve injury, tricuspid regurgitation, valve repair

## Abstract

Traumatic tricuspid regurgitation (TTR) is an uncommon cause of acute right ventricular dysfunction. The surgical approach can be complex, and repair tends to have a lower success rate when right heart failure symptoms are present. We present a case of a 56-year-old man with chronic isolated tricuspid valve flail and severe TTR due to high-energy blunt chest trauma from a vehicle accident 24 years prior. This report emphasizes the critical role of echocardiography in diagnosing trauma-related primary tricuspid regurgitation and the necessity of early detection to facilitate surgical intervention before irreversible damage happens.

## Introduction

Traumatic tricuspid valve regurgitation is rare, often secondary to blunt chest trauma, and most seen in road traffic accidents [1,2]. The aortic valve is the most commonly affected, followed by the mitral valve and then the tricuspid valve [3]. The absence of acute physical signs and the presence of other traumatic injuries can delay the diagnosis, thus underestimating the actual prevalence of traumatic tricuspid regurgitation (TTR) [4,5]. It is not uncommon to diagnose post-traumatic tricuspid regurgitation after several months or even years [6,7]. The advent of echocardiography has allowed easier diagnosis and therefore early intervention before right ventricle (RV) dysfunction which worsens the prognosis [8].

We report the case of a patient with TTR complicated with RV dysfunction diagnosed several years after a car crash.

## Case presentation

A 56-year-old man, with no prior valvular heart disease and a history of a road traffic accident dating back to 1998, was brought to the emergency department, where he was treated for a double injury: lower limb fracture and a chest blunt trauma. Sadly, no echocardiogram was done at the time, hence no cardiac abnormality was detected. Eight years later, he reported a stage II NYHA dyspnea related to a moderate tricuspid valve regurgitation that had been demonstrated on routine transthoracic echocardiography. The patient received diuretics and remained clinically stable. Thereafter, the patient presented to the cardiology department in 2021, with progressively worsening dyspnea and recurrent acute decompensated right heart failure.

**Figure 1:**
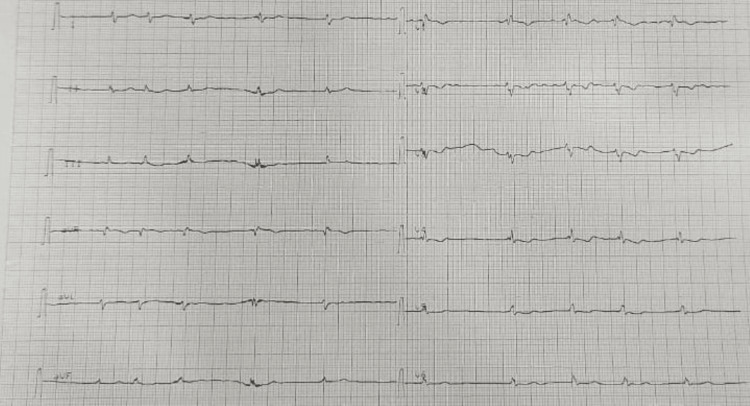
EKG of the patient

**Figure 2:**
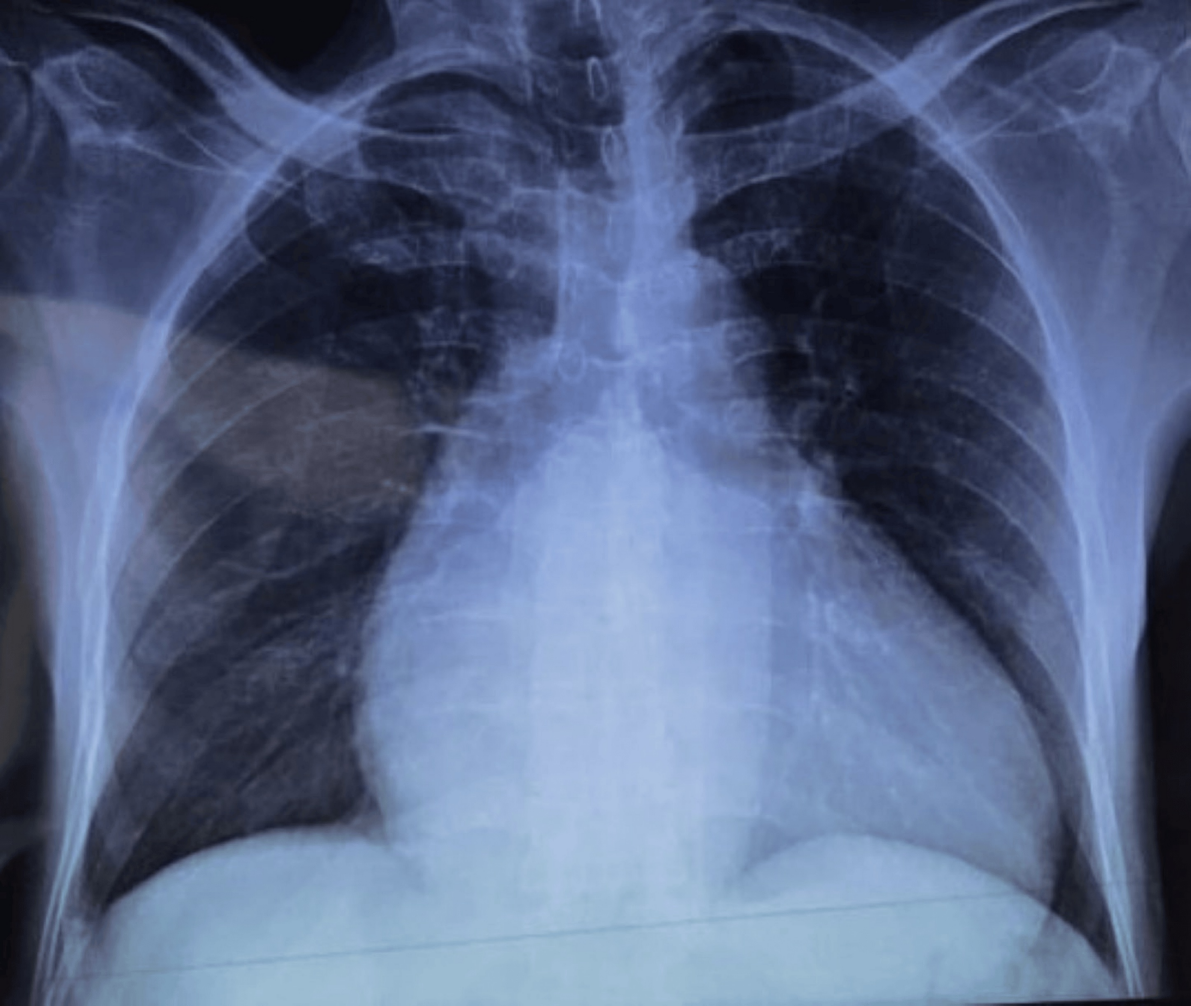
X-ray frontal view of the patient

Physical examination found a systolic murmur at the lower right edge of the sternum, jugular venous distension, hepatomegaly, and swelling of the lower limbs. The electrocardiogram (EKG) showed atrial fibrillation, right axis deviation, and incomplete right bundle branch block (Figure 1). The chest X-ray shows cardiomegaly with a cardiothoracic report (CTR) of 0.6 and a right overhang (Figure 2).

**Figure 3:**
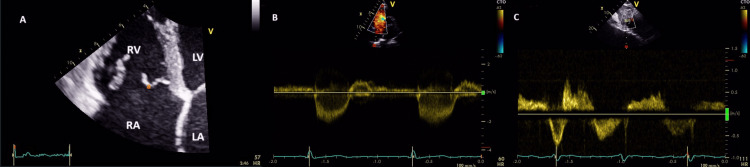
Transthoracic echocardiogram images A: Four-chamber view showing a dilated RV (right ventricle) and prolapse of the tricuspid septal leaflet (a); B: laminar tricuspid flow; C: reversal of the systolic flow in the hepatic veins showing the extent of the tricuspid regurgitation RA: right atrium, LA: left atrium, LV: left ventricle

Transthoracic echocardiography showed massive laminar TR caused by septal valve prolapse on chordae tendineae rupture, a very dilated RV (diameter: 63 mm) with an altered systolic function, ectatic right atrium (RA), dilated tricuspid annular (52 mm), and dilatation of inferior vena cava (IVC) (42 mm), with no other associated valve damage (Figure 3).

The first time, the patient refused any surgery and received only medical treatment (furosemide + spironolactone). As his clinical profile worsened, he finally accepted the intervention in 2022.

After midline sternotomy, surgical exploration showed a very dilated right ventricle (RV) with very altered contractility. A CEC was installed between an aortic cannula and two cavity cannulas.

**Figure 4:**
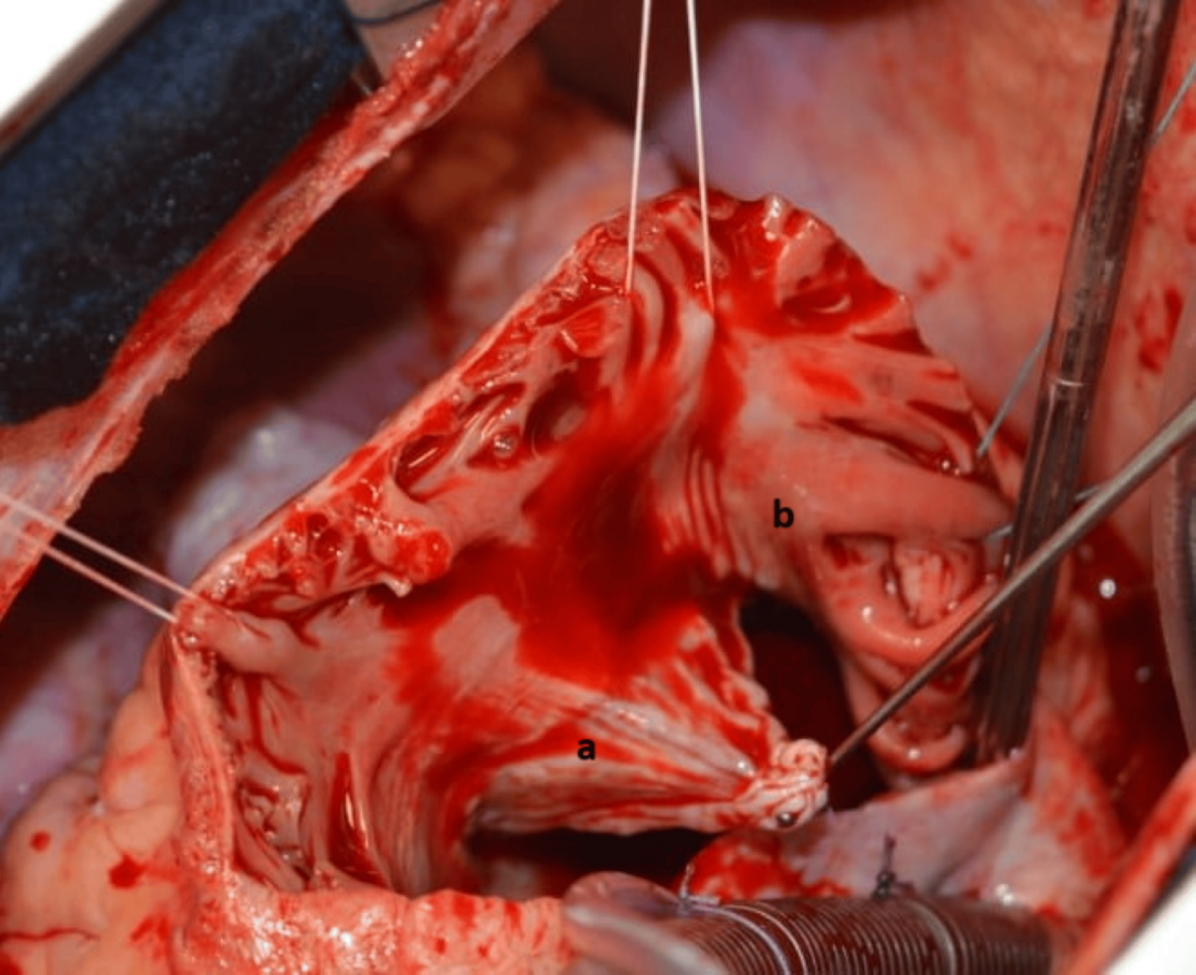
Surgical view showing retracted anterior tricuspid valve Anterior tricuspid valve (a); right atrium (b).

**Figure 5:**
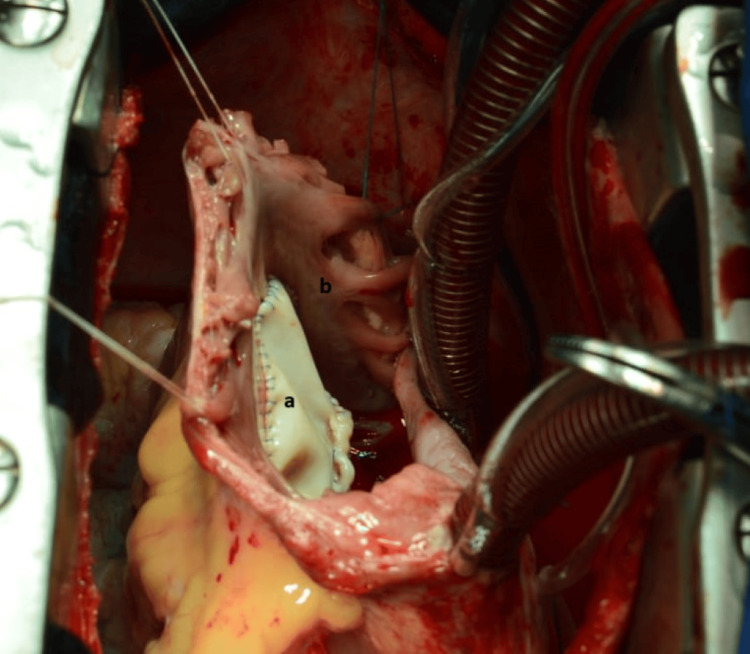
Surgical view showing a leaflet augmentation technique The anterior leaflet is first detached from the anteroseptal to the anteroposterior commissure and then enlarged by the use of a bovine pericardial patch (a); right atrium (b).

**Figure 6:**
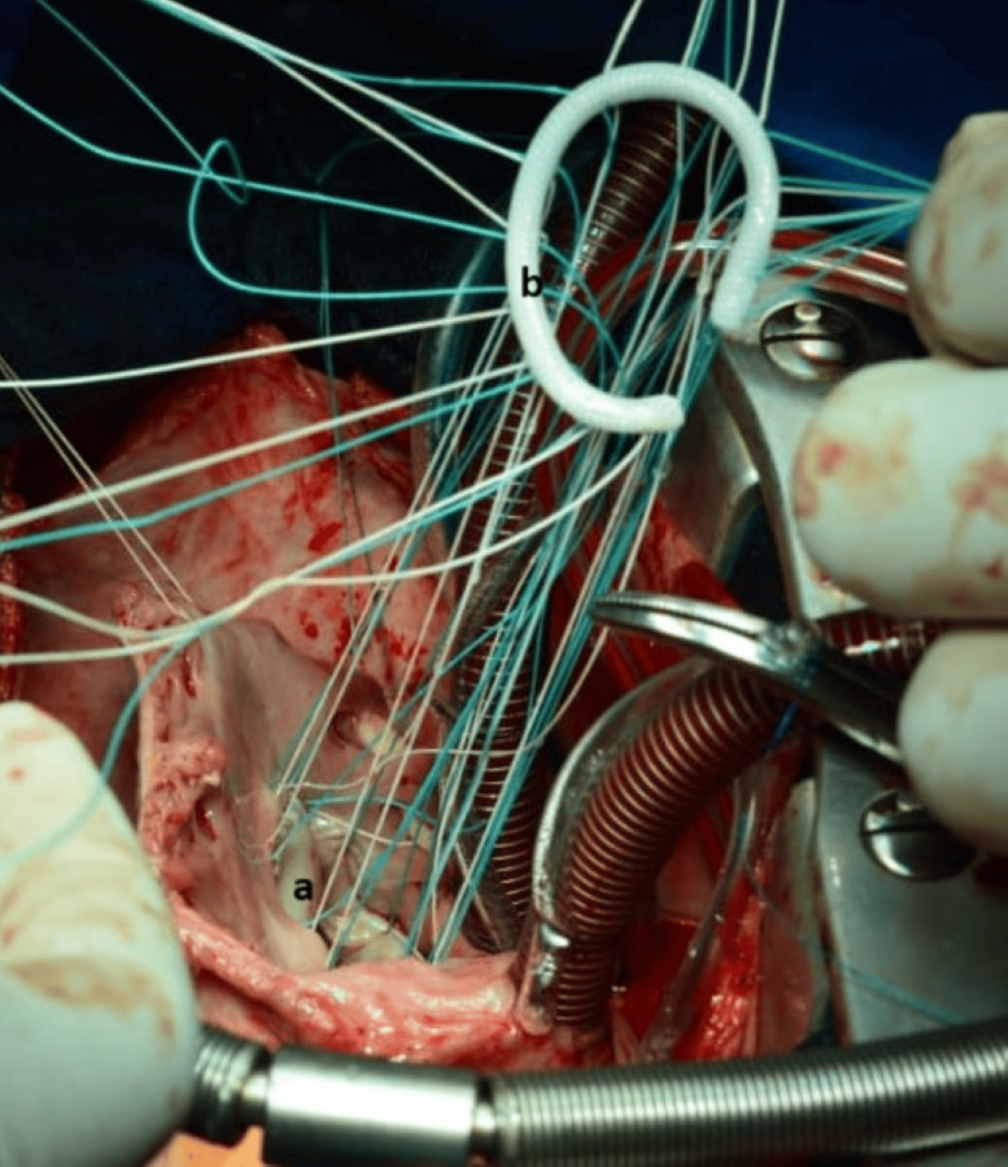
Prosthetic tricuspid ring annuloplasty Bovine pericardial patch (a); tricuspid ring (b)

Intraoperative exploration of the tricuspid valve revealed prolapse of the septal valve after rupture of the main cord and retracted anterior valve (Figure 4). It was decided to enlarge the anterior valve with a heterologous pericardial patch (7x7 cm) (Figure 5) and then insert the ruptured cord on the free wall of the RV with a Gore-Tex No.5/0 thread along with an annuloplasty by Edwards No. 34 ring (Figure 6).

After the surgical procedure, the serum test was satisfactory. After weaning from cardiopulmonary bypass, transesophageal echocardiography revealed trivial TR. Yet, the patient presented with hemodynamic instability 24 hours later, warranting the realization of a TTE that showed significant TR with severe RV dysfunction. We decided to take the patient back to the operating room.

After right atriotomy, we detected a prolapse of the septal valve following a rupture of the pillar to which the notochordal was attached. As a result, tricuspid valve replacement was performed with Sorin No. 33 (Sorin Group, Saluggia, Italy), a mechanical prosthesis. The patient improved hemodynamically.

Nevertheless, on postoperative Day 4, he presented with oliguria lasting 48 hours with impaired renal function (urea = 2.85 g/l, creatinine= 58 mg/l) and hyperkalemia at 6.8 mmol/l. On postoperative Day 6, the patient underwent a hemodialysis session but soon experienced hemodynamic instability that degenerated into a refractory cardiogenic shock, ultimately leading to death.

## Discussion

TTR is an uncommon complication resulting from blunt chest trauma, associated with significant mortality and morbidity [9]. It was first documented by Williams in 1829, and the first surgical correction was performed by Cooley in the late 1950s [2]. Although TTR is rare, representing only 0.02% of traumatic injuries, it may be underdiagnosed due to the nonspecific and often tolerable nature of its symptoms [4,10]. In recent years, the incidence of TTR seems to be rising, likely due to advances in echocardiography and an increase in traffic accidents globally [11].

The pathophysiology of TTR involves a severe and sudden increase in heart pressure due to the simultaneous compression of the right ventricular outflow tract and closure of the tricuspid valve during the transition from end-diastole to early systole. This pressure spike creates significant strain on both the valvular and subvalvular structures [11,12]. The right ventricle's location immediately behind the sternum makes it particularly vulnerable to deceleration forces during blunt trauma. The most commonly reported injuries are rupture of the chordae tendineae, followed by rupture of the anterior papillary muscle and tears in the anterior leaflet of the tricuspid valve [13].

Zhang et al. studied 10 patients with traumatic tricuspid regurgitation. Among them, seven had anterior chordal rupture, two had anterior papillary muscle rupture, and one had both anterior chordal and anterior leaflet rupture. Five patients underwent tricuspid valve repair, while the others had valve replacement. Post-surgery, all patients were classified as NYHA Class I, with no instances of moderate or severe tricuspid valve regurgitation. Additionally, all patients remained symptom-free and experienced no other cardiovascular events during follow-up [14]. Richard et al. described nine patients with traumatic tricuspid regurgitation, primarily due to anterior papillary muscle rupture. In this cohort, valve repair was performed in only one case, with the remaining eight patients requiring tricuspid valve replacement [15].

Maisano et al. reviewed 74 reported cases and found that chordal rupture was the most common cause of tricuspid regurgitation (41 cases, 55.4%), with anterior chordal rupture accounting for 75.6% of these cases. Papillary muscle rupture was the second most common cause (20 cases, 27.0%), followed by leaflet rupture (11 cases, 14.8%) [16].

Ma et al. reported on 13 patients with traumatic tricuspid regurgitation. The average time from trauma to diagnosis was 37.4 months, and the average time from trauma to surgery was 54.4 months. During surgery, anterior chordal rupture was identified in eight patients, anterior papillary muscle rupture in three, a combination of anterior papillary muscle and chordae rupture in one, and an anterior leaflet defect in one. Seven patients had annular dilation. Valve repair was successfully performed in all 13 patients [8].

The clinical manifestations of TTR depend on the severity of the valve injury. Due to the right ventricle’s compliant nature, TR may be well-tolerated in the early stages [17]. When symptoms do occur, they can include palpitations, dyspnea, or chest pain, though these symptoms can be nonspecific in the context of chest trauma [18]. Patients might remain asymptomatic for extended periods and present later with heart failure, which can complicate eventual repair [18,19]. In our case, the interval between trauma and surgery was 24 years.

Unlike the mitral and aortic valves, the tricuspid valve is a complex structure; even though 2D echocardiography is the first bedside tool recommended for chest trauma patients, especially if cardiac injury is suspected, it doesn’t seem to provide sufficient information to assess the morphology and mechanism of TTR. On the other hand, 3D imaging permits a simultaneous display of all three tricuspid valve leaflets, providing a surgical view of the tricuspid valve, which allows surgeons to plan the surgical procedure prior to entering the operating room. It can be performed in the operating room to obtain further anatomical details prior to the actual surgery. If transthoracic echocardiography is inconclusive, transesophageal echocardiography should be considered, especially if cardiac involvement is strongly suspected or in the case of blunt chest trauma with chest injuries [20].

Currently, surgical experience with this valve disease is limited to case reports and small series involving fewer than 25 patients [4]. Surgical indications are not well-defined [21] and depend on the patient's symptoms and the specific anatomical lesions [22]. Treatment strategies for tricuspid regurgitation include valve repair or valve replacement, with valve repair generally being preferred. However, valve repair is not always successful and should be customized to address the specific valvular pathology [23,24]. For a flail leaflet, repair options include plication with or without resection, replacement of chordae with neochordae, and reconstruction of papillary muscles [8,19]. Other techniques discussed in the literature include anchoring the anterior leaflet with ruptured chordae tendineae to the septal leaflet edge-to-edge (double-orifice repair) as described by Alfieri and colleagues [24]. Annuloplasty is a crucial aspect of nearly all mitral and tricuspid valve repairs, as it corrects annular dilatation, enhances leaflet coaptation by reducing the annulus size, decreases tension on suture lines, and helps prevent future annular dilatation [25]. In traumatic tricuspid regurgitation, the presence of multiple lesions, progressive dilation, deterioration of the right ventricle, and the often contracted state of the chordae tendineae and affected leaflets generally make traditional valve repair techniques impractical [24], In our case, we employed multiple repair techniques. Van Son et al. reported on 13 patients with traumatic tricuspid insufficiency who all underwent surgical treatment. Valve repair was deemed feasible in five cases (38%), while the remaining eight patients (62%) required valve replacement [13].

Maisano et al. [16] reported six cases of severe post-traumatic tricuspid regurgitation treated using conventional reconstructive surgical techniques, including artificial chordae implantation, papillary muscle reinsertion, and commissuroplasty.

Alfieri et al. described five patients with severe traumatic tricuspid regurgitation who underwent valve reconstruction using the Clover technique. This approach involves stitching the midpoint of the free edges of the tricuspid leaflets together to create a clover-shaped valve. The study found the Clover technique to be a straightforward, rapid, and effective method for correcting TTR. It is particularly useful for complex tricuspid valve lesions and cases with right ventricular enlargement or dysfunction resulting from long-standing chronic volume overload [24].

TTR is a serious and progressive condition, making early diagnosis crucial [22]. Indications for early surgery include severe or torrential regurgitation, especially when accompanied by clinical and echocardiographic signs of right ventricular strain. Valve repair is preferred, but valve replacement is an alternative if repair is not feasible. Delaying surgery can result in right ventricular failure, which complicates repair [3,26,27]. Additionally, a delay may lead to fibrosis of the valve and subvalvular apparatus, making repair even more difficult [13]. For these reasons, early referral for surgical intervention is recommended and often leads to better outcomes. Ma et al. noted this in a case series of 13 patients [8]. Zhang et al. demonstrated that early surgical intervention for TTR is recommended to enhance the possibility of valve repair and prevent right heart failure [14].

## Conclusions

This case emphasizes the importance for physicians to be vigilant about potential cardiac complications after blunt chest trauma and to use echocardiography as an initial diagnostic tool. While many patients may manage well for years despite traumatic tricuspid regurgitation, early diagnosis and surgical intervention can help prevent right ventricular deterioration and make tricuspid valve repair more feasible.

## References

[1] Avegliano G, Corneli M, Conde D, Ronderos R (2014). Traumatic rupture of the tricuspid valve and multi-modality imaging. Cardiovasc Diagn Ther.

[2] Parmley LF, Manion WC, Mattingly TW (1958). Nonpenetrating traumatic injury of the heart. Circulation.

[3] Meel R, Ngutshane B, Gonçalves R, Mogaladi S (2020). A case of severe tricuspid regurgitation related to traumatic papillary muscle rupture. Case Rep Cardiol.

[4] Gayet C, Pierre B, Delahaye JP, Champsaur G, Andre-Fouet X, Rueff P (1987). Traumatic tricuspid insufficiency. An underdiagnosed disease. Chest.

[5] Croxson MS, O'Brien KP, Lowe JB (1971). Traumatic tricuspid regurgitation. Long-term survival. Br Heart J.

[6] Schuster I, Graf S, Klaar U, Seitelberger R, Mundigler G, Binder T (2008). Heterogeneity of traumatic injury of the tricuspid valve: a report of four cases. Wien Klin Wochenschr.

[7] Jin HY, Jang JS, Seo JS (2011). A case of traumatic tricuspid regurgitation caused by multiple papillary muscle rupture. J Cardiovasc Ultrasound.

[8] Ma WG, Luo GH, Sun HS, Xu JP, Hu SS, Zhu XD (2010). Surgical treatment of traumatic tricuspid insufficiency: experience in 13 cases. Ann Thorac Surg.

[9] Lee JW, Song JM, Park JP, Lee JW, Kang DH, Song JK (2010). Long-term prognosis of isolated significant tricuspid regurgitation. Circ J.

[10] Ismailov RM, Weiss HB, Ness RB, Lawrence BA, Miller TR (2005). Blunt cardiac injury associated with cardiac valve insufficiency: trauma links to chronic disease?. Injury.

[11] Lin SJ, Chen CW, Chou CJ (2006). Traumatic tricuspid insufficiency with chordae tendinae rupture: a case report and literature review. Kaohsiung J Med Sci.

[12] Bertrand S, Laquay N, El Rassi I, Vouhé P (1999). Tricuspid insufficiency after blunt chest trauma in a nine-year-old child. Eur J Cardiothorac Surg.

[13] van Son JA, Danielson GK, Schaff HV, Miller FA Jr (1994). Traumatic tricuspid valve insufficiency. Experience in thirteen patients. J Thorac Cardiovasc Surg.

[14] Zhang Z, Yin K, Dong L, Sun Y, Guo C, Lin Y, Wang C (2017). Surgical management of traumatic tricuspid insufficiency. J Card Surg.

[15] Richard P, Vayre F, Sabouret P, Gandjbakhch I, Ollivier JP (1997). Outcome of traumatic tricuspid insufficiency, treated surgically. Apropos of 9 cases [article in French]. Arch Mal Coeur Vaiss.

[16] Maisano F, Lorusso R, Sandrelli L, Torracca L, Coletti G, La Canna G, Alfieri O (1996). Valve repair for traumatic tricuspid regurgitation. Eur J Cardiothorac Surg.

[17] Emmert MY, Pretre R, Suendermann S, Weber B, Bettex DA, Hoerstrup SP, Falk V (2011). Severe traumatic tricuspid insufficiency detected 10 years after blunt chest trauma. Clin Res Cardiol.

[18] Jonjev ŽS, Milosavljević AM, Bjeljac I, Todić M, Koruga S (2018). Tricuspid valve avulsion 3 years after blunt chest trauma. J Card Surg.

[19] Hirao S, Minakata K, Sakaguchi H, Watanabe K, Yamazaki K, Sakata R (2016). Surgical repair of tricuspid regurgitation due to annular detachment caused by chest trauma. J Cardiol Cases.

[20] Cheng Y, Yao L, Wu S (2017). Traumatic tricuspid regurgitation. Int Heart J.

[21] Bonow RO, Carabello BA, Kanu C (2006). ACC/AHA 2006 guidelines for the management of patients with valvular heart disease: a report of the American College of Cardiology/American Heart Association Task Force on Practice Guidelines (writing committee to revise the 1998 Guidelines for the Management of Patients With Valvular Heart Disease): developed in collaboration with the Society of Cardiovascular Anesthesiologists: endorsed by the Society for Cardiovascular Angiography and Interventions and the Society of Thoracic Surgeons. Circulation.

[22] Messika-Zeitoun D, Thomson H, Bellamy M (2004). Medical and surgical outcome of tricuspid regurgitation caused by flail leaflets. J Thorac Cardiovasc Surg.

[23] Baraki H, Saito S, Al Ahmad A, Fleischer B, Haverich A, Kutschka I (2015). Beating heart versus arrested heart isolated tricuspid valve surgery. Int Heart J.

[24] Alfieri O, De Bonis M, Lapenna E, Agricola E, Quarti A, Maisano F (2003). The “clover technique” as a novel approach for correction of post-traumatic tricuspid regurgitation. The. Journal of Thoracic and Cardiovascular Surgery.

[25] Gillinov AM, Cosgrove DM 3rd, Shiota T (2000). Cosgrove-Edwards Annuloplasty System: midterm results. Ann Thorac Surg.

[26] Longfellow E, Aberle C, Lamelas J (2022). Traumatic injury of the tricuspid valve-navigating the challenges in diagnosis and management. J Cardiothorac Vasc Anesth.

[27] Pink K, Qu Y (2022). Chronic flail tricuspid valve related to blunt chest trauma: a case report. Cureus.

